# The Endocannabinoid System and Physical Exercise

**DOI:** 10.3390/ijms24031989

**Published:** 2023-01-19

**Authors:** Daniela Matei, Dan Trofin, Daniel Andrei Iordan, Ilie Onu, Iustina Condurache, Catalin Ionite, Ioana Buculei

**Affiliations:** 1 Department of Biomedical Sciences, Faculty of Medical Bioengineering, University of Medicine and Pharmacy “Grigore T. Popa” Iasi, 700454 Iasi, Romania; 2 Doctoral School of the Faculty of Medicine, University of Medicine and Pharmacy “Grigore T. Popa” Iasi, 700454 Iasi, Romania; 3 Department of Individual Sports and Kinetotherapy, Faculty of Physical Education and Sport, “Dunărea de Jos” University of Galati, 800008 Galati, Romania; 4 Center of Physical Therapy and Rehabilitation, “Dunărea de Jos” University of Galati, 800008 Galati, Romania; 5 Doctoral School of the Faculty of Chemical Engineering and Environmental Protection “Cristofor Simionescu”, Technical University “Gheorghe Asachi” Iasi, 700050 Iasi, Romania

**Keywords:** the endocannabinoid system, physical exercise, CB1, CB2, runner’s high

## Abstract

The endocannabinoid system (ECS) is involved in various processes, including brain plasticity, learning and memory, neuronal development, nociception, inflammation, appetite regulation, digestion, metabolism, energy balance, motility, and regulation of stress and emotions. Physical exercise (PE) is considered a valuable non-pharmacological therapy that is an immediately available and cost-effective method with a lot of health benefits, one of them being the activation of the endogenous cannabinoids. Endocannabinoids (eCBs) are generated as a response to high-intensity activities and can act as short-term circuit breakers, generating antinociceptive responses for a short and variable period of time. A runner’s high is an ephemeral feeling some sport practitioners experience during endurance activities, such as running. The release of eCBs during sustained physical exercise appears to be involved in triggering this phenomenon. The last decades have been characterized by an increased interest in this emotional state induced by exercise, as it is believed to alleviate pain, induce mild sedation, increase euphoric levels, and have anxiolytic effects. This review provides information about the current state of knowledge about endocannabinoids and physical effort and also an overview of the studies published in the specialized literature about this subject.

## 1. Introduction

The pathological mechanisms underlying cardiovascular diseases are very well defined these days; oxidative stress, inflammation, and endothelial dysfunction are the main factors involved. Physical exercise (PE) is recognized as an important component of a healthy lifestyle, and the recommendation is 30 min of exercise training per day with moderate intensity, which is considered to reduce the risk of cardiovascular events [1].

We all know that moderate PE when performed regularly can decrease blood pressure, LDL cholesterol, and body mass index as well as increase HDL cholesterol, endothelial function, and insulin sensitivity. Moreover, moderate physical activity has a positive impact on the body because it helps maintain the health of bones, muscles, and joints. During this type of effort, the level of free radicals produced is moderate and the antioxidant capacity increases, making the body adapt.

In contrast, subjects exposed to large amount of physical activity have impaired cardiovascular health. Overload training may induce intense oxidative stress and can damage proteins, glucose, lipids, and nucleic acids in cells. In aerobic exercise, the reactive oxygen species are produced during mitochondrial respiration, but in anaerobic exercise, oxidative stress may result from the ischemia/reperfusion cycle of muscle contraction and/or immune system responses following muscle damage [2]. Furthermore, intense training may lead to inflammation that contributes to the formation of a vicious circle contributing to increase oxidative stress.

Developing the habit of regularly performing physical activity positively impacts long-term biological parameters related to body weight and has associated benefits, thus leading to reducing mortality and even ameliorating pain. Considering this association, the exercise capacity is relevant as a better mortality predictor compared to other risk factors associated with cardiovascular conditions [3].

The last two decades have shown an increase in efforts made by the scientific community towards understanding the mechanisms involved in maintaining an active healthy lifestyle. This would eventually lead to the development and implementation of programs that rely on physical exercise to enhance neuroplasticity, help recover motor deficits, or even prevent illness in such a sedentary lifestyle environment that we live nowadays. Hawkes demonstrated that PE can contribute to the natural production of endorphins (endogenous opioid neuropeptides) that can play a role in relieving pain and inducing wellbeing [4].

This wide range of neurobiological rewards induced by physical training, such as the sense of wellbeing, decrease in pain, and reduction in anxiety, was referred to as “runners high”. Dietrich and McDaniel argued that “endorphin theory” is unsustainable because the effects of β-endorphins on PE are unclear due to the fact that the “exercise endorphin connections” are ambiguous [5].

The amino acid sequence of β-endorphin is almost identical to the sequence of the adrenocorticotrophic stress hormone, which increases in PE, leading to serious detection problems. However, the most serious limitation of “endorphin theory” is the measurement of β-endorphins from the bloodstream, as it cannot be considered an indicator of central effects due to the endorphins being too large to properly cross the blood–brain barrier (BBB) [5].

Since beta endorphins may not efficiently cross the BBB, peripheral activation in the systemic circulation is less likely to cause any changes within the brain. Therefore, while endorphins are released during exercise and can stop peripheral muscle soreness that results from exercise, they cannot be the source of the runners’ high [5,6,7,8]. Most of the currently available data came from a study about RH experience. For many years, it was believed that RH was only induced by endorphin release; however, new results suggest a crucial role played by the endocannabinoid system [9,10,11]. Running increases plasma levels of β-endorphin and anandamide in mice and men [12,13]. Fuss et al., in 2015, showed that cannabinoid receptors mediate acute anxiolysis and analgesia after running. They showed that anxiolysis depends on intact cannabinoid receptor 1 (CB1) receptors found on forebrain GABAergic neurons and that pain reduction depends on the activation of peripheral CB1 and CB2 receptors in mice [7].

Currently, researchers have turned their attention to endogenous endocannabinoids as the true source of analgesia, sedation, anxiolysis, and reduced depression in endurance exercisers [5,14,15].

Siebers et al., in 2021, recruited 63 healthy participants and had them run at moderate-intensity levels on a laboratory treadmill for 45 min; after that, the same participants walked for 45 min. The researchers found that running vs. walking led to higher plasma levels of endocannabinoids and that participants “exhibited increased euphoria and decreased anxiety after 45 min of running on a treadmill in a moderate-intensity range compared to walking.” Researchers randomly used an opioid-receptor antagonist (naltrexone) to block opioid receptors, because they wanted to investigate if euphoria and reduced anxiety levels were dependent on opioids. Participants who were given the opioid-receptor blocking naltrexone still experienced exercise-induced euphoria and anxiolysis [15]. The findings of this study indicate that RH in humans does not depend on opioid signaling but depends on endocannabinoids, similar to the results obtained in mice [15].

## 2. Physiology of the Endocannabinoid System

Anandamide (AEA), an endocannabinoid (eCB), is a fatty acid neurotransmitter that can cross the blood-brain barrier readily, and has two main molecular targets in the form of cannabinoid CB1 and CB2 receptors [5]. AEA and 2-arachidonylglycerol (2-AG), the two most studied eCBs, are released by neurons both centrally and peripherally in an activity-dependent manner to modulate synaptic activity and plasticity [10].

Till date, five types of eCB have been recognized. Beside the two AEA and 2-AG, three more eCBs were found, i.e., 2-arachidonylglycerol ether, virodhamine, and N-arachidonyldopamine (NADA). The endocannabinoid system is a complex system made up of cannabinoid receptors, endogenous ligands, specific proteins involved in the endocannabinoid biosynthesis, and degradation enzymes like fatty acid amide hydrolase (FAAH) [16].

### 2.1. The ECS and the Nervous System

The CB1 receptor is mainly expressed in the brain in areas of primary sensory and motor regions as well as areas of memory, cognition, and emotion, all of which overlap the areas of the autonomic nervous system and neuroendocrine system [17]. Kano et al. suggested that the highest levels of CB1 receptor binding were observed in the olfactory bulb, as well as in the regions of the dentate gyrus, the lateral striatum, the globus pallidus, the entopeduncular nucleus, the substantia nigra (SN) pars reticulata, and the cerebellar molecular layer [18]. Moderate levels were found in the frontal, parietal, and cingulate regions, in the amygdala, in the ventromedial hypothalamus, in the parabrachial nucleus, in the nucleus of the solitary tract, and in the spinal dorsal horn [18]. Low levels of CB1 receptor were found at the level of the thalamus and at the level of the brain stem nuclei [18]. The CB1 receptor is also expressed in peripheral nerve terminals, and in non-neuronal cells, such as endocrine, liver, lung, kidney, endothelial, myocardium, and skeletal muscles; white and brown adipose tissues; glial cells; and astrocytes [19]. As neuromodulators, CB1 receptors modulate synaptic transmission of many neurotransmitters, such as GABA, glutamate, serotonin, acetylcholine (Ach), or cholecystokinin (CCK), among others [20].

The endocannabinoid system is believed to play a major role in neuropathic pain (NP). NP, besides pain, is characterized by sensory impairment that can sometimes lead to extreme phenomena such as allodynia or hyperalgesia. The symptomatology may extend towards affecting cognitive and emotional functions. In spite of all these, spontaneous pain is the main burdensome aspect of NP [21]. The ECS modulates pain control and pathological aspects of NP by the activities of CB1 and CB2 receptors [21]. Peripherally, in nociceptive terminations, CB1R inhibits the nociceptive stimuli and also inhibits neurotransmitter release and pain transmission at the dorsal root ganglia and the dorsal horn of the spinal cord. At the supraspinal level, they inhibit the ascension of nociceptive transmission, especially towards the thalamus. They play a role in activating the descending inhibitory pathway by inhibiting GABA release in the medulla, as well as influencing the emotional pain perceived in the limbic system. In contrast, CB2R was more involved in modulating immune responses associated with chronic pain in the spinal cord [21,22,23,24,25].

Although the mechanisms described above are important for understanding the functionality of the ECS in terms of developing novel therapeutic approaches to treat pain, especially related to receptor agonists, it is of interest to search for possible self-regulatory ways in which the endocannabinoids can help without exogenous intervention. Therefore, the sudden euphoria experienced by endurance sport practitioners is believed to enhance hypoalgesia and even sedation in addition to increasing euphoric levels and reducing anxiety [5,15]. Moreover, euphoria and increased eCBs relate differently to exercise intensity, duration, and individual aspects [26]. Apparently, blocking the opioid receptors with central opioid blockers does not inhibit exercise-dependent hypoalgesia, as opposed to CB1 and CB2 receptors blockade, thus suggesting the eCB signaling role [7,27].

The CB2 receptor can be found mainly in immune cells such as macrophages, B lymphocytes, and blood stem cells as well as in the organs of the immune system, e.g., spleen, tonsils and thymus and to a lesser extent, in the cerebral cortex, cerebellum, and the gastrointestinal tract [28,29]. Recent studies suggested that CB2 receptors are expressed in microglia or glial cells [29]. In the gastrointestinal tract, CB2 receptors regulate intestinal inflammatory reactions [30]. Evidence of a CB3 receptor or “anandamide receptor” in the brain and in the endothelial tissues is still under evaluation [28].

Both the CB1 and CB2 receptors are coupled to Gi/o protein alpha-subunits that inhibit adenylyl cyclase activity and, thereby, depending on the cell type, either inhibit voltage gated calcium channels or activate potassium channels [28,31]. The effect of CB1 activation is neuronal inhibition [28].

The CB2 receptor is an important target for neurodegenerative diseases and cancer; the new radioligand [18F]LU13 is a promising radioligand for the imaging of upregulated CB2R expression using positron emission tomography under pathological conditions in the brain [32]. 

**Figure 1:**
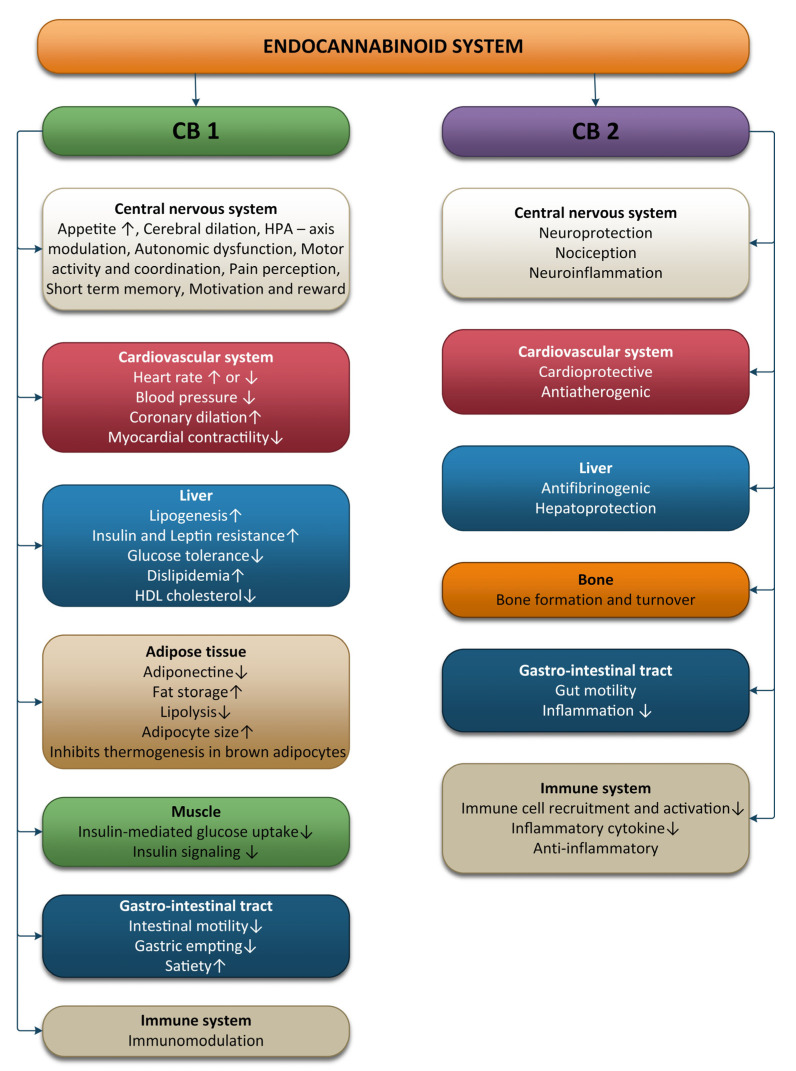
Effects of the endocannabinoid system on different systems and organs.

The ECS is involved in various processes, including brain plasticity, learning and memory, neuronal development, nociception, inflammation, appetite regulation, digestion, metabolism, energy balance, motility, and regulation of stress and emotions (Figure 1).

The ECS modulates the sympathetically driven hypothalamic-pituitary-adrenocortical (HPA) axis as well as the hypothalamic-locus coeruleus-norepinephrine axis. In response to biological stress, the first neurons activated are the NE norepinephrine neurons of the locus coeruleus. Subsequently, the paraventricular nucleus (PVN) of the hypothalamus initiates corticotropin releasing hormone (CRH) secretion, thus inducing adrenocorticotropin hormone (ACTH) release. ACTH stimulates the release of glucocorticoids (cortisol in humans) from the adrenal cortex into general circulation. The amygdala activates the HPA axis, whereas the hippocampus and prefrontal cortex inhibit the HPA axis [33].

Sympathetic nerve terminals contain CB1R, and activation of these receptors has been shown to inhibit norepinephrine release and reduce pain. The SNS mediates the anxiety-like effects observed after CB1R blockade [34]. CB1R activity limits hypothalamic CRH release and restricts ACTH and glucocorticoid release [35]. In contrast, glucocorticoids induce rapid increases in endocannabinoid synthesis in brain areas that shape the perception of psychological stressors [36]. After acute stress, the released glucocorticoids activate G-protein membrane receptors in the basolateral amygdala (BLA), promoting a rapid increase in retrograde 2-AG signaling that leads to the suppression of GABAergic synaptic inputs onto BLA principal neurons, inducing fast increases in anxiety-like behavior [37]. Enhancing FAAH activity results in glucocorticoid release and stress-related anxiety-like behavior [38]. Fatty acid amide hydrolase inhibition or costimulation of CB1R and TRPV1 (transient receptor potential cation channel subfamily V member 1) receptors decreases the release of stress-induced corticoids [39,40].

Stress produces bidirectional changes in the AEA and 2-AG, with stress exposure reducing AEA levels and increasing 2-AG levels in the hypothalamus, prefrontal cortex and hippocampus; these changes are associated with a decreased HPA-axis activity that modulates memory processes and pain perception after stressful stimuli [41].

Prolonged stress may influence the overall health via several different pathways, i.e., alterations in the autonomic nervous system (increased SNS and decreased PNS activity), in the neuroendocrine activity as well as in the immune, behavioral, and cognitive functions. Chronic stress causes hyperactivity of the sympathetic nervous system, which occurs with reduced activation of the HPA axis. Straub et al. termed this physiological phenomenon “uncoupling of SNS and HPA-axis” [42].

The autonomic imbalance during chronic stress described by increased SNS and suppressed PNS activity is associated with cellular stress, including an increase in the levels of cytokine and reactive oxygen species. Exposure to chronic stress reliably causes a downregulation or loss of CB1R in brain regions related with emotional processing. Chronic stress promotes FAAH activity through glucocorticoid stimulation, and anandamide levels are maintained low in the hippocampus, hypothalamus, PFC, and amygdala, which leads to hyperexcitability of the amygdala circuits [43]. The inhibition of FAAH activity prevents these effects of chronic stress and reduces changes that chronic stress promotes in BLA [44,45]. The amygdala sends CB1R-positive projections to the bed nucleus of the stria terminalis (BNST), which is connected with the ventral tegmental area and the locus coeruleus. Glutamatergic and GABAergic projections from the amygdala to the BNST are each sufficient for the development of anxious responses to unpredictable stimuli [43]. CB1R in cortical glutamatergic terminals inhibit glutamate release, and their activation induces anxiolysis, whereas CB1R activation from GABAergic neurons of the forebrain restricts the inhibitory GABA tone and mainly facilitates anxiety [43].

The vagus nerve has afferent and efferent fibers controlling cardiopulmonary, gastrointestinal, metabolism, and many other functions. Vagal afferent neurons transmit visceral information to the nucleus of the solitary tract (NTS) and release glutamate controlled via cannabinoid-sensitive excitatory transient receptor potential (TRP) channels [46]. Three groups of PNS afferent fiber types can be determined using the conduction velocity: slow-conducting unmyelinated C-fibers, moderate-conducting thinly myelinated Aδ-fibers, and fast-conducting heavily myelinated A-fibers. All these afferent subgroups abundantly express a complement of TRP channels, although Transient Receptor Potential Vanilloid Type 1 (TRPV1) is selectively expressed on all C- and some Aδ-fibers, but not in A fibers [46]. TRPV1 is activated by AEA, which reduces synthesis of 2-AG and is implicated in hyperalgesia, chronic pain, inflammation, neurogenesis, and anxiety [47,48]. The sensitivity of TRPV1 to AEA requires CB1-mediated activation of phosphoinositide-3-kinase and phospholipase C [49]. TRPV1 is responsive to both capsaicin and AEA, but TRPV1 requires CB1 activation for sensitivity to AEA but not capsaicin [50].

The activation of vagal afferents via the TRP channels by ECS may have the same therapeutic effects such as electrical vagus nerve stimulation [51,52]. Many studies have shown that acute cannabis use causes hypertension, tachycardia, and a fivefold increase in risk of adverse cardiovascular events including stroke and heart attack [53,54,55,56]. In contrast, chronic cannabis use prevents hypertension, atherosclerosis, and improves cardiovascular disease outcomes [53,54,55,56]. These observations highlight that acute consumption of cannabis leads to harmful effects, because it causes impaired vagal afferent signaling, while the chronic effects of cannabis are associated with increased vagal afferent signaling. These different actions can be explained by the desensitization between TRP channels and CB1 receptors [57]. TRP channels are expressed in primary sensory afferents such as vagal afferents to central circuits, and in non-neuronal tissue such as smooth muscle [58].

Enhancement of vagal afferent signaling may serve to ameliorate autonomic dysfunction associated with a variety of other chronic diseases such as atherosclerosis, diabetes, obesity, epilepsy, anxiety, and depression [53,54,55,56,59,60,61,62]; all of these are each associated with vagal hypofunction, increased heart-rate, and heart-rate variability. Both VNS and ECS increase may improve autonomic dysfunctions by stimulation of vagal afferents that compensate for vagal hypofunction associated with these disorders. The mechanisms by which the vagus may influence these chronic diseases are by reducing oxidative stress and systemic inflammation [63]. Oxidative stress is reduced with the help of antioxidant enzymes (superoxide dismutase, catalase, and glutathione peroxidase, etc.) and non-enzymatic antioxidants (melatonin, lipoic acid, Coenzyme Q10, glutathione, uric acid, bilirubin, etc.). Exercises, depending on the intensity can induce ROS generation, which will result in increased activity of enzymatic antioxidants, thereby leading to increased resistance to oxidative challenges [64,65]. The progenitor cells in the hippocampal region in adults and the embryonic cells produce endocannabinoids, which are expressed upon stimulation of CB1 receptors and the suppression of FAAH enzyme [66]. This demonstrates the involvement of the ECS in neurogenesis. Moreover, the ECS possesses neuroprotective functions, providing protection against acute hypoxia, oxidative stress, and traumatic insults. In mice with a closed head injury, the levels of 2-AG were elevated. Further administration of 2-AG exogenously diminished edema in the brain, decreased the infarct volume, prevented death of hippocampal neuronal cells, and facilitated a better clinical recovery [67].

### 2.2. The ECS and the Cardiovascular System

CB1 receptors are well represented at the level of the cardiovascular system. CB1 can be found in the myocardial cells, coronary artery, endothelial cells, and smooth muscle cells. CB2 was also detected in the coronary endothelial cells, smooth muscle cells, and myocardial cells. This means endocannabinoids are produced at this level, are circulating through blood, and play an important role in the management and regulation of the cardiovascular system functions [68]. The results of studies conducted on animals show that endocannabinoids have an impact on blood pressure and heart rate, but the effects are influenced by the condition of the animal (conscious or anesthetized); this implies that endocannabinoids act by modulating the autonomic nervous system [69]. Anandamide has an extended response to hypotension in animals that are anaesthetized, thereby inhibiting the sympathetic activity on the nerve terminals present at the level of the heart and vasculature. CB1 receptors upon activation in arteries inhibit the release of noradrenaline in the mesenterial arterial bed region [69]. In conscious rats, anandamide produces bradycardia with a transient condition of hypertension followed by long-lasting pressor effects, which are accompanied by renal and mesenteric vasoconstriction [70]. Upon activation of CB1, adrenaline blood levels increase via adrenergic beta-2 receptors as well as increased sympathetic activity.

Furthermore, the ECS is involved in the central control of blood pressure associated with the brainstem baroreceptor complexes [71]. Anandamide microinjection can prolong the reflex inhibition of the renal sympathetic nerve activity and suggest that inhibition of the GABAergic tone can lead to an increased sensitivity to baroreflexes. Moreover, an increase in blood pressure due to phenylephrine can enhance the concentrations of anandamide in the region of the nucleus tractus solitarius and support the ECS in controlling and regulating the activity of baroreflexes [72].

A study conducted on mice with blocked CB1 receptors showed significant conditions of elevated heart rate and blood pressure due to irregular breathing during sleep and a disturbed sleep-awake cycle [68]. Studies conducted on rats with hypertension showed that CB1 receptor expression was higher in the cardiac tissue and in the aortic endothelium of rats compared to healthy rats [73].

### 2.3. The ECS and the Immune System

The ECS also has an important role in the maintenance of immune homeostasis. Immune cells, especially immune B cells, followed by monocytes, natural killer cells, neutrophils, and CD4 and CD8 lymphocytes, express ECS receptors. CB2 receptors are 10–100 times more present at this level [68,74,75]. Both CB1 and CB2 receptors are important modulators of the immune system, inducing immunosuppression [76].

CB2 receptors have a modulatory role on the immune system, which is related to the induction of apoptosis, suppression of cell proliferation, inhibition of pro-inflammatory cytokines production, increase in anti-inflammatory cytokines, and induction of regulatory T cells [77]. CB2 activation induces a shift from Th1 to Th2 immune response and induced myeloid-derived suppressor and T-regulatory cells [78]. A deficiency of CB2 receptors can activate neutrophils to the inflammation sites. The activity of macrophages associated with tumors is also believed to be inhibited by CB2 receptors. The cytokine implicated in the activity and differentiation of T cells is interleukin 2, which is secreted upon the activation of natural killer cells and T-cells. AEA has a high affinity to CB1, thereby reducing the production of pro-inflammatory IL-6 [79]. Sardinha et al., in 2014, pointed out that the inhibition of MAGL and FAAH, the enzymes that respectively degrade 2-AG and AEA, has CB2-mediated anti-inflammatory effects [80].

Classically activated macrophages (M1) have pro-inflammatory and anti-tumor properties by releasing various types of pro-inflammatory cytokines and chemokines such as TNF, IL-6, and IL-1β. Alternative activated macrophages (M2) perform anti-inflammatory and immunosuppressive effects by releasing anti-inflammatory cytokines (IL-10) and promote tumor progression. An imbalance of M1/M2 is responsible for inflammation [81].

CB2 receptors inhibit immune cell activation and pro-inflammatory mediator release. Moreover, CB2 receptor stimulation with its selective agonists reversed these pathological conditions by reducing both B and T lymphocytes, by promoting anti-inflammatory activities, and by limiting pro-inflammatory cytokine release in macrophages, thereby inhibiting M1 polarization [82,83]. Immunosuppression is the consequence of stimulation of CB2 receptors, which helps to reduce inflammation and prevents associated injury in tissues.

### 2.4. The ECS and the Digestive System

One of the best described axes of the body is the bidirectional axis that links the brain and the gastro-intestinal tract (GI); this axis is formed of hormonal and neuronal pathways and is known as the brain-gut axis. The nutrient intake and availability as well as the energy status at the peripheral level are supervised by the GI tract, liver, adipose tissue, pancreas, and skeletal muscle, all of which provide information to the CNS with the aim of maintaining energy homeostasis. The vagus and splanchnic nerves are responsible for the neural signaling of this axis. The communication between the liver and gut, pancreas, and adipose tissue is also ensured by the vagus nerve. Areas of the brain that are stimulated by gut hormones are areas were the blood-brain-barrier is absent or were transporters for the gut hormones that permitted them to signal the CNS. These areas are represented in the brainstem by the area postrema and in the hypothalamus by the arcuate nucleus. Information from the enteric nervous system and from the enteroendocrine cells are transmitted by the vagal afferent neurons to the brainstem in the nucleus of the solitary tract [84].

The ECS is involved in the peripheral and central control of gut functions. Endocannabinoids are not stored like hormones or neurotransmitters at the level of vesicles; moreover, specific signals are needed to induce their synthesis at the lipid membrane level, making them ideal mediators for responding in real-time to the ever-changing feeding state of an organism. The enzymes involved in the synthesis of anandamide, FAAH, and NAPE-PLD can be found in the GI tract, and CB1 and CB2 receptors are also present in the GI tract in the enteric nerves and epithelium. CB1 is highly present in the enteric nervous system on all classes of enteric neurons except inhibitory motor neurons and on some enteroendocrine cells. CB2 is also found on enteric neurons and on immune and epithelial cells in the GI tract [85]. When AEA and 2-AG trigger CB1 and CB2 receptors, the biochemical responses will frequently depend on the type of cell stimulated; the response varies from decreased levels of cAMP thought the inhibition of Ca2+ channels and adenylate cyclase activity to increased activity of mitogen-activated protein kinase pathways, phospholipases, and K+ channels [86].

Normally, under physiological conditions, the ECS activity is ensured by the CB1 receptor, whose activation triggers actions such as increased gastrointestinal motility, mesenteric vasodilatation, and suppression of fluid and acid secretion. Because of the high expression of CB1 in neurons of the CNS, this receptor is also implicated in the regulation of peripheral organs that play an important part in metabolic homeostasis and also control feeding, reward, and energy expenditure. When inflammation is present in the GI tract, the activation of both receptors takes place, resulting in the synthesis of anti-inflammatory cytokines with a role in reducing inflammation and its secondary damages [87,88,89,90,91,92].

Appetite and food intake are locally balanced by the activation of the CB1 receptor that modulates the activity of hypothalamic neurons that cause the release of orexigenic and anorexigenic neuropeptides as well as the function of the mesolimbic and brainstem neurons by directing information to these neurons from the periphery. Therefore, CB1 receptors are implicated both in the homeostatic and hedonic aspects of food intake [86].

The white adipose tissue (WAT) is an essential regulator of energy storage and systemic metabolic homeostasis, and studies conducted in vitro show that the ECS plays a role in the regulation of adipogenesis and lipogenesis, with the CB1 receptor being expressed at the level of mature white adipose cells. The activation of glucose uptake, lipoprotein lipase, and fatty acid synthase; stimulation of PPARg expression and adipogenesis; inhibition of cAMP release, AMPK, and mitochondrial biogenesis; and inhibition of adiponectin production in hypertrophic adipocytes are the mechanisms described in the regulation of adipogenesis and lipogenesis. It was observed that, in animal and human obesity, the CB1 receptor expressed at the level of white adipocytes, WAT AEA, and 2-AG levels are usually deregulated [13,86,92,93,94,95].

The CB1 receptor plays an important role in lipogenesis regulation by enhancing the production of acetyl-Coenzyme A (CoA) and Fas, because it upregulates the sterol regulatory element binding transcription factor 1 (SREBF1). Endocannabinoids and the ECS receptor are also produced in muscle cells and express the enzymes implicated in ECS synthesis and degradation [86].

## 3. Physical Exercise and the ECS

Raichlen et al. hypothesized that cursorial mammals and humans possess highly specialized biological mechanisms that facilitate long-distance running. Dogs and humans showed an increase in AEA levels as a response to endurance exercise of 30 min running or walking on the treadmill as compared to ferrets (a non-cursorial species). The 2-AG levels, however, were not modified [10]. The following year, Raichlen et al. proved that only endurance exercise at 70–80% of age-adjusted maximum heart rate (AAMHR) influences AEA levels. The 10 subjects involved in the study had to walk or run during 4 days for 30 min on the treadmill at four different intensities. The walking speed or the high-intensity running parameters did not influence the AEA as compared to the mentioned intensity [95].

Sparling et al. described the ECB activation by physical exercise. A total of 24 male subjects performed either running or cycling at a 70 to 80% maximum heart rate for 45 min. Compared to control participants who remained seated for 50 min, the AEA levels increased for the active group, suggesting that AEA acts on the peripheral sensory fibers to reduce pain [13].

Similar results were obtained by Heyman et al., in a group of 11 cyclists, who had to pedal at 55% of the maximal power output (Wmax) followed by a 75% Wmax. The results suggested that the AEA levels only increased after increased endurance exercise, and still continue to rise after recovery. The 2-AG levels were not modified whatsoever [96].

Crombie conducted a study in 2018 with the aim of examining the interaction between the endogenous opioid and endocannabinoid systems in a pain modulation process known as exercise-induced hypoalgesia. A total of 58 subjects were analyzed, who were given an opioid antagonist (naltrexone) and a placebo before performing pain testing and isometric exercise. 2-AG and 2-oleoylglycerol (2-OG) increased after exercise for both the naltrexone and placebo lots, while AEA and oleoylethanolamine (OEA) levels increased only in the placebo group. However, no notable differences were observed with palmitolethanolamine (PEA) between the groups. Because pain reductions were observed under both conditions, the results suggest that the opioid system may not be the primary system involved in exercise-induced hypoalgesia and that 2-AG and 2-OG may contribute to nonopioid exercise. The data suggest, among other facts, that the opioid system might not be primarily implicated in exercise-induced hypoalgesia [27].

Animal models with specific disease characteristics have been developed in studies trying to determine possible therapeutic approaches. A peripheral lesion initially leads to hypersensitivity of the affected area. Then, inflammation occurs, with the central sensitization eventually leading to allodynia by aberrant responses. In this process, microglia and astrocytes in the dorsal horn add up to the appearance of nociceptive responses and spinal hyperexcitability, as the pain becomes chronic [97,98].

Endocannabinoids are generated as a response to high intensities of activity. They can act as short-term circuit breakers, generating antinociceptive response for a short and variable period of time. The theory of ECs as endogenous analgesics becomes more complex as the components involved in their expression are also located in inhibitory neurons, immune cells, and glial cells among the CNS. Even so, in the equation of pain resolution, it seems that so far, ECs are also elevated in chronic pain [99].

Paszcuk et al. started from the known facts of antinociceptive effects of cannabinoid receptors on neuropathic pain in rodent experiments, trying to provide evidence of pain modulatory activity in brachial plexus avulsions (BPA). The subject is important and needs to be investigated, as in humans, the prevalence of brachial plexus injuries is increasing due to the increase in car accidents and practice of extreme sports, as well as obstetrical traumatology. The impact is huge in terms of the morbidity and the quality of life of people worldwide. In the study, mice received either BPA or sham surgery. By using RT-PCR and immunohistochemistry, mRNA levels and protein expression of CB1R and CB2R were investigated, while glial cell activation, MAP kinases, and transcription factors were assessed via immunohistochemistry. The authors used cannabinoid agonists, administered on the 5th and 30th day after the surgical procedure. The treatment reduced allodynia at both endpoints post-surgery, but especially at the 30th day. It also prevented the expression of glial cells and MAK kinases and also enhanced transcription factor activation [100]. This represents a step towards the development of future analgesic treatments specially designed for specific types of nervous lesions.

Da Silva Santos et al. studied the pain induced by efforts a widely debated phenomenon worldwide. The review aims to provide a detailed description of exercise-induced analgesia, which was beneficial for the relief of pain caused by various conditions, such as fibromyalgia, low back pain, neuropathy, and osteoarthritis. The analyzed studies demonstrated that both during and after exertion, different endogenous systems are activated that release substances or neurotransmitters, such as opioids, nitric oxide, serotonin, catecholamines, and endocannabinoids, which can modulate the perception of pain. Moreover, starting from the premise that physical exercise releases analgesic substances, all that remains is to closely investigate the exercise modality and at what fitness level the analgesia occurs. This can help to develop effective strategies for using physical exercise for the treatment of different types of pain, which could eventually lead to a reduced need for pharmacological drugs [101].

In 2014, Galdino et al. conducted a study that investigated the involvement of the endocannabinoid system in the antinociception induced by resistance exercise (RE). An experiment was carried out on male Wistar rats that were subjected to acute resistance exercises. The nociceptive threshold was measured using a mechanical nociceptive test (paw pressure) before and after exercise. Before starting the test, the rats were injected with enzyme-inhibiting cannabinoid receptor inverse agonists and an anandamide reuptake inhibitor. At the end of the test, cannabinoid receptors were quantified from rat brain tissue using Western blotting and immunofluorescence. In addition, endocannabinoid plasma levels were measured via isotope dilution chromatography-liquid mass spectrometry. RE-induced antinociception was prevented by the pre-injection with CB1 and CB2 cannabinoid receptor inverse agonists. In contrast, the pre-administration of the metabolizing enzyme inhibitors and the anandamide reuptake inhibitor prolonged and enhanced this effect. RE also produced an increase in CB1 cannabinoid receptor expression and activation in rat brain tissue and in the dorsolateral and ventrolateral periaqueductal regions as well as an increase in endocannabinoid plasma levels. The study suggests that a single session of resistance exercises activates the endocannabinoid system, thus inducing antinociception [102].

Runner’s high is an ephemeral feeling some sport practitioners experience during endurance activities, such as running. The release of eCBs during sustained physical exercise appears to be involved in triggering this phenomenon. The last decades have been characterized by an increased interest in this emotional state induced by exercise that is believed to alleviate pain, induce mild sedation, increase euphoric levels, and have anxiolytic effects.

With respect to the interactions between dopamine and cannabinoids, Boecker et al. proposed a study with positron emission tomography ‘‘ligand activation’’ by using 6-O-(2-[18F]fluoroethyl)-6-O-desmethyldiprenorphine ([18F]FDPN) as an opioidergic ligand. The study was performed in 10 athletes at rest and after 2 h of running a distance of approximately 20 km. The reduction in the availability of the opioid receptor was evidenced in the prefrontal and limbic/paralimbic brain as the level of euphoria was elevated after running and inversely correlated with opioid binding in the anterior cingulate cortex, bi and parainsular cortex, as well as in the temporoparietal regions. This suggests the implication of the frontolimbic brain regions in mood regulation [9].

Other studies, such as the one conducted by Fernandes et al., suggest the implication of the adipose hormone leptin. The signaling of the leptin receptor (LepR) would activate transcription-3 (STAT3), especially in dopamine neurons among the ventral tegmental area (VTA). In their experiment design, mice lacking STAT3 in dopamine neurons exhibited greater voluntary running; therefore, STAT3 deletion increased the reward-relevant behavior, whereas intra-VTA leptin blocked the rewarding effect. This brings insight towards the motivational effects of running via Lepr-STAT3 modulation of dopamine that might increase stamina and also result in better adaptation to endurance activities [103].

According to Fuss and Gass, endocannabinoids may influence the intention for voluntary physical activity (such as running) in rodents, and even seem to influence the total motion distance via CB1 receptors. Nevertheless, in this study, the impact upon the modifications of emotional behavior related to voluntary exercise appears to be less significant [11].

The emotional component of physical exercise, especially by practicing endurance sports, can in some cases, be desired by obtaining the runner’s high emotion, and even possibly be related to long-term improvement of mood, according to Steptoe and McDaniel [5,11,104,105].

In mice with deleted CB1 receptors, less voluntary running activity was noticed, indicating the ECS implications in the brain reward networks [84,86]. Moreover, voluntary running can be compared to addictive patterns, as CB1 receptor blockade or deletion reduces reward driven activities in mice [11,106,107].

In the study designed by Siebers et al., 64 endurance healthy sports enthusiasts (32 men, 32 women, 18–50 years old) performed 50 min sessions of treadmill running and walking. They were monitored after receiving naltrexone (a central opioid blocker) or placebo; no CB1 or CB2 blockers were available at the moment of performing the study. Euphoria and reduction in anxiety were the main items assessed by monitoring the plasmatic levels of eCB-like molecules palmitoyl ethanolamide and arachidonic acid before and after the exercise. Anxiety was evaluated using a virtual reality environment. A questionnaire regarding the runner’s high experience was completed by each participant. The study proved that the opioid blockade did not reduce anxiety or increase euphoric states. It also proved that eCBs correlate with runner’s high, increases euphoria, and has anxiolytic effects. A rather natural environment could be much more useful for designing studies in future for evaluating runner’s high, as opposed to laboratory conditions [108].

Running may be a better way of increasing eCB levels, followed by cycling, when running is performed at an intensity of 70–85% of AAMHR. This is usually accompanied by an increase in AEA and leads to benefits related to anxiety and mood, especially related to experience with the exercise performed, and sometimes, nature-like environment [108].

This is the context in which Feuerecker et al. studied the specific impact of a natural environment setting in the functioning of the eCB system. Twelve healthy male subjects were monitored while hiking in the Alps under hypoxic conditions, at two different altitudes. AEA increased more at higher altitudes; nevertheless, these environmental conditions alone did not influence the control group (static, taken to the same altitude by helicopter). Similar to the results of other studies regarding the ECS, the 2-Ag levels were not modified [109].

Alongside the neurobiological effects that can mediate well-being induced by exercise, the ECS might also be involved in enhancing neurogenesis related to physical activity. Most available studies have been performed in animal models (rodents). The results on neurogenesis available at this moment show controversial aspects. Neurogenesis in the dentate gyrus after 2 months of moderate alcohol consumption appears to be similar to that observed after running, in the same manner in which withdrawal effects were observed after ceasing regular running in mice, all of which seems to resemble mood disorders in sedentary people [106,110].

In spite of the anxiolytic and antidepressant benefits of voluntary physical activity, there is a possibility that different environmental setups could influence various results, leading to sometimes inconclusive neurogenesis findings [11]. Reduced neurogenesis in CB1 deficient mice does not seem to influence the results of the forced swim test, a well-known test for its antidepressant effect, and also in voluntary wheel running, according to Dubreucq et al. [105]. This can only suggest an independent effect of the ECS in the process of neurogenesis [11].

Running reduces anxiety in normal mice, suggesting a possible increase in neurogenesis. CB1-altered subjects experienced reduced anxiety patterns correlated with decreased neurogenesis. Nevertheless, neither the altered CB1 subjects nor the controls showed modifications in anxiety after wheel running and both showed an increase in neurogenesis by 40% [105]. In contrast, excessive running seems to increase neurogenesis by 100%, and correlates with modifications in mice with anxious behavior [11]. Transgenic procedures performed to alter hippocampal neurogenesis actually increase anxiety behavior [111].

The extent of physical exercise, as well as perceptions of wheel exposure, can influence neurogenesis, especially as the use of the wheel can lead to stressful, stereotypic or addictive, or even rewarding behavior, thus influencing neurogenesis [11,112].

Further studies in mice will aim towards being be able to clarify the accurate physiological mechanisms by which the ECS is involved in neurogenesis, as well as the emotional connections implied. Only then, specific types of physical activities will be designed for humans in order to enhance therapeutic neuroplasticity. Until then, just as Fuss and Gass conclude, one may only hope for an enjoyable runner’s high experience [11].

Loprizi et al. investigated the mode of action of maximal and minimal physical exercises as a function of episodic memory. To date, the mechanisms of this effect have been attributed to changes in long-term potentiation, neurotrophic production, angiogenesis, and neurogenesis. The authors described a complementary mechanical model that suggests that the endocannabinoid system can influence, to some extent, the effects of physical exercises on memory. Both the role of the ECS on memory function and the role of physical exercise on endocannabinoid changes were considered. The research carried out should represent a starting point in the analysis of the role of physical exercise on memory function [113].

Meyer aimed to describe the acute changes in serum endocannabinoids in a prescribed moderate-intensity exercise session and a self-selected/preferred-intensity exercise session in women with major depressive disorder, and at the same time, to determine the relationships between changes in endocannabinoid concentration and mood states. At the end of the study, it was found that moderate-intensity physical exercises increased the number of EC [114].

In 2019, Jaromin et al. used an animal model to test a hypothesis that the evolution of increased aerobic exercise performance may be facilitated by the evolution of motivation to undertake physical activity, mediated by the brain ECS. The study provided information supporting the hypothesis that the increase in endocannabinoid concentration plays an important role in increasing aerobic performance [115].

Studies report that physical activity correlates with increased serum endocannabinoid concentrations and increased expression of the CB1R in the brain. This fact leads to positive neurological effects, including antidepressant effect, improvement in memory, development of neuroplasticity, and reduction in neuronal inflammation [116].

Crombie et al. studied the effect of physical effort on the anxiety state of patients. The mechanisms responsible for the anxiolytic effects of exercise are not fully understood, although recent studies suggest that the ECS plays an important role. The study used a randomized approach to examine anxiety and fear ratings of predictable or unpredictable electric shock administration, circulating endocannabinoid concentrations, and mood states immediately after moderate-intensity aerobic exercise (30 min on a treadmill at 70–75%). The results of the study suggest that aerobic exercise exerts psychological benefits in patients with anxiety, due to increases in circulating endocannabinoid concentrations induced by exercise [117]. Fifteen subjects had to cycle for 30 min at 70% VO2max (moderate intensity), 30 min at 80% VO2max (high intensity), or rest for 30 min. Prior and after the 30 min task, they were analyzed using fMRI while performing a four fingers sequence of keypresses. Thus, memory was investigated for motor sequences, revealing an important increase of AEA after both high and moderate exercises. The rise of AEA levels correlated with expansion of the caudate nucleus and increase in hippocampal activity [118].

Using the alarming figures of the high incidence of mental illness provided by the WHO, Amatriain-Fernández et al., carried out in 2021, a review that debates the fact that the ECS could be considered a modulator, a fact explained by the positive results of exercise for the management of mental disorders. From a clinical point of view, this promising area could be exploited by targeting elements of the ECS in order to increase the benefits of physical exercise for patients with mental illness [119].

A growing body of evidence suggests the triangulation of the ECS, exercise, and neurological health. The study carried out by Forteza aims to correlate the functional interactions between aerobic exercise and molecular and cellular pathways related to endocannabinoids, in the hypothalamus, hippocampus, and periphery, paying special attention to associations with emotional state, cognition, and mental health. It has also been pointed out that gut microbiota imbalance, which affects the gut-brain axis and metabolism, also influences certain inflammation pathways modulated by the eCBome. The integrity of the gut microbiota could thus be crucial for the emergence of neuroinflammation and mental states. Since the launch of this hypothesis, the interest of researchers has been directed towards how exercise modulation of the peripheral eCBome affects brain functions; thus, key elements could be discovered for the prevention and treatment of neuropsychological disorders [120].

In vivo animal studies and clinical trials have shown existing links between overactivity of the ECS and various diseases. Metabolic disorders seem to be related to higher circulating levels of endocannabinoids, with obesity being the most commonly studied disease. The activity of ECS receptors seems to be increased by obesity, and in return, this will cause an intensification of lipogenesis and higher food intake with an increase of fat storage [121]. Insulin resistance, type 2 diabetes, and dyslipidemia seem to also be connected to the dysregulation of the ECS, specifically with the stimulation of the CB1 receptor at the level of insulin-dependent tissues (skeletal muscles, liver, or adipose tissue). Moreover, glucose and lipid metabolism can be increased when CB1 activity is suppressed [122,123].

Researchers have started to show interest in the way this connection between PE and the ECS can be used for the treatment of metabolic disorders. Currently, only a few studies on this subject can be found in the specialized literature.

Di Marzo et al. conducted a study on 49 viscerally obese men who for 1 year were included in a lifestyle modification program (regular physical activity and healthy diet) and showed that the effects on the ECS were decreased levels of AEA and 2-AG and the metabolic effects were decreased body weight, waist circumference, and visceral tissue, associated with increased levels of HDL3-C [124]. Another study by You et al. studied the same subject but on 30 overweight and obese women who underwent a 20-weeks program of moderate (45–50% of maximum heart rate) or intense exercise (70–75% of maximum heart rate) associated with caloric restriction. The results showed increased expression of cb1r gluteal adipose tissue gene and decreased expression of FAAH abdominal adipose tissue related to the impact that PE and caloric restriction had on the ECS. The metabolic results were decreased glucose and insulin and decreased body weight [125]. Fernandez et al. also performed a study on 77 obese women who performed normal daytime activity for the first 6 days of the study and then performed moderate-vigorous physical activity for the following 6 days. The effects of the moderate-vigorous PE translated in higher plasma levels of AEA and OEA and decreased BMI and waist circumference [126].

The results of the studies conducted on rats showed the impact of different types of PE, mainly treadmill running, with different intensity and duration on lipid levels, weight, fasting plasma glucose, etc., in relation with the ECS activity. Yan et al. studied male Wistar rats that performed 1 h of swimming 3 days a week for 6 months, and the results showed lower body weight, blood pressure, and visceral adipose tissue associated with decreased expression of CB1R in the VAT and SAT and increased expression of PPARδ in the VAT [127]. Ju et al. evaluated C57Bl/6J male mice that performed 1 h of treadmill running, 6 times a week for 6 weeks, and observed a decrease in body weight, body fat percentage, and LDL-C and TG levels as well as an increase in HDL-C levels in association with decreased NAPE-PLD expression in the brain and epididymal fat, decreased plasma AEA and 2-AG, and decreased CB2R expression in the epididymal fat and CB1R and CB2R expression in the brain [128]. Decreased body weight and fasting plasma glucose levels were found to be connected to the modification of the ECS in male Wistar rats that performed 1 h of treadmill running (70–80% MAV) for 12 weeks, 5 times a week in two studies conducted by Gamelin et al. [129,130].

Due to the very high prevalence of metabolic diseases at the global level, the methods of control and prevention were and are currently being studied. Physical activity being accessible to any person, regardless of their socioeconomic status, is a method that must be considered and studied more carefully. The studies in the field, although not in a very large number, show how PE acts on the ECS by influencing existing metabolic imbalances or preventing their occurrence.

Physical activity can have both acute and chronic impacts on endocannabinoid mediators, as can diet. As part of a study carried out in 2022, Forteza followed the influence of diet on the peripheral response to maximal acute aerobic exercise. A sample of active adult women without underlying metabolic conditions was analyzed. The impact of the 7-day standardized Mediterranean diet (MedDiet) and the control diet inspired by the Canadian macronutrient intake (CanDiet) on the endocannabinoidome and short-chain fatty acid metabolites after maximal aerobic exercise was compared. In general, plasma endocannabinoid levels, their congeners, and some polyunsaturated fatty acids increased significantly after maximal aerobic exercise. At the end of the exercise, they recovered their initial values within 1 h. Most N-acylethanolamines and polyunsaturated fatty acids showed increased levels directly after exercise when participants consumed the MedDiet, but not when they consumed the CanDiet. This impact was different for monoacylglycerol endocannabinoid congeners, which in most cases responded similarly to acute exercise during MedDiet or CanDiet treatment. The conclusion of this study emphasizes that endocannabinoid mediators respond to acute maximal aerobic exercise in a manner that depends on the diet consumed in the week prior to exercise [131].

Schonkel et al. conducted a study designed to highlight the paradoxical overlap in the context of metabolic health and skeletal muscle response to acute and chronic aerobic exercise and resistance exercise in relation to the ECS by using a meta-analysis tool. The conclusion of the study was that physical exercise changes the gene expression of cannabinoid receptors and the enzymes involved in the biosynthesis and breakdown of the ECS. In addition, endocannabinoids are possibly involved in regulating the immune response to exercise, thus modulating communication between the skeletal muscle and other tissues [132].

Not all available studies have shown an increase in AEA post-exercise, similar to the results published in 2016 by Cedernaes et al. In 16 male subjects performing 30 min physical exercise (75% VO2 reserve capacity), no rise in AEA levels was detected. The participants were monitored in two periods: the first after three nights with 8 h of sleep, and the other, after 3 nights of only 4.25 h of sleep (opportunity of sleep). However, OEA increased in both situations, unlike the release of 2-AG in the sleep-deprived session [133]. No modifications in AEA levels were observed by Stone et al. However, they revealed some interesting aspects. Instead of sports enthusiasts or amateur athletes, nine choir women were evaluated while being involved in 30 min of dancing, singing, reading, or spinning. OEA, and not AEA or PEA, increased after spin class, considering the heart rate frequency less than 70% AAMHR. In this case, the participants in the group were not familiarized with either spinning or dancing. An increase in the ECS was surprisingly noticed during singing, thus suggesting that positive emotions or experiences can release endocannabinoids; therefore, other activities can also be considered instead of endurance sports [134].

**Figure 2:**
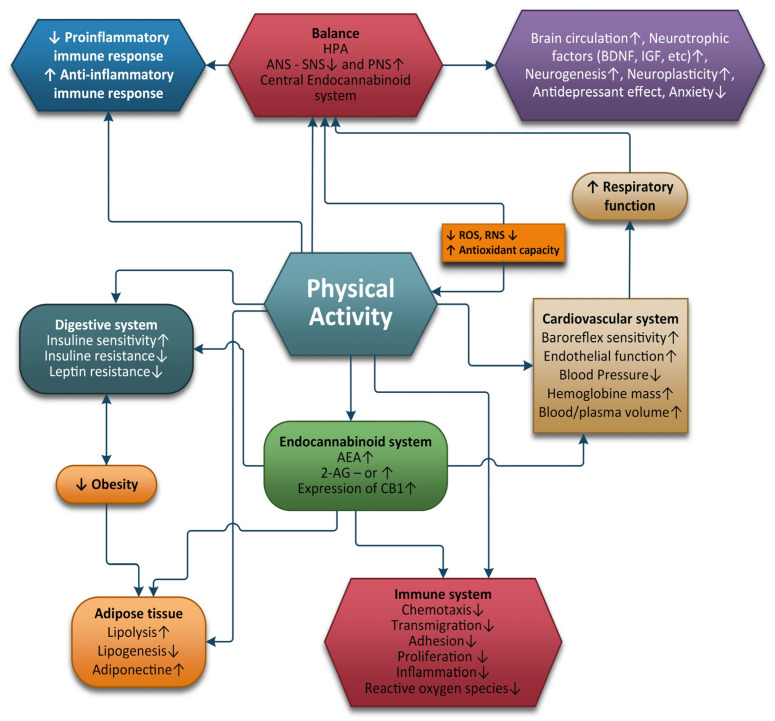
Positive effects of physical activity.

Figure 2 shows the positive effects that physical activity has on different systems and organs and on the ECS.

The response to acute stress is a normal process of adaptation of the body to the changing conditions of the environment that tries to maintain a stable internal environment. In acute stress, the AEA level decreases, leading to the appearance of anxiety. Simultaneously, the 2-AG levels increased, which reduces the perception of pain and activates memory for remembering and avoiding the danger in the future. If the stress became chronic, the 2-AG levels remained at high levels, which stimulate CB1 receptors in the brain, so the brain compensates by decreasing its CB1 receptors. Decreased eCB receptors can generate a negative emotional state, which can lead to major depression [41]. PA of moderate intensity exercise can raise blood levels of AEA and 2-AG. The runner’s high can result in pain reduction, reduced anxiety, and stimulating a state of well-being and happiness. Regular exercise can also maintain a healthy weight, reduce inflammation, increase antioxidants, decrease ROS, balance the autonomic nervous system, and manage stress levels through the HPA axis, all of which contribute to the maintenance of the ECS balance.

Adherence to a physical activity program should be optimal in order to achieve benefits from activating the ECS. The routine is, however, intriguing to understand when it comes to moods. Brellenthin et al. assessed 36 individuals by establishing three groups according to their weekly physical activity routines: low, moderate, and high. On the first day, they had to run on the treadmill within 70–75% VO2max, while on the following day, they could run at their own preferred intensity. The levels of AEA, 2-AG, OEA, and PEA significantly increased in both situations. In contrast, the levels of OEA and AEA were higher only on the first day. The weekly physical activity did not impact the eCB. Moreover, accordingly to the questionnaires taken regarding depression, anxiety, or anger, the overall mood improved better in the prescribed conditions [135].

Persistent pain leads to disability and poor quality of life, and is the leading cause for years lived with disability [136,137,138,139].

Medicinal cannabinoids are currently used to treat multiple pain conditions, such as back pain and cancer-related pain [140]. Despite the increased demand for cannabinoids among individuals with persistent pain, the evidence is deemed low, or very low, for analgesic efficacy [140].

Gedin et al., in 2022, performed a systematic review and meta-analysis to evaluate the magnitude of the placebo response found in double-blind randomized clinical trials in which cannabinoids, cannabis, and cannabis-based medicine were compared with placebo for the treatment of clinical pain. Twenty studies, including 1459 individuals (mean [SD] age, 51 [7] years; age range, 33–62 years; 815 females [56%]), were included. Pain intensity was associated with a significant reduction in response to the placebo, with a moderate to large effect size (mean [SE] Hedges g, 0.64 [0.13]; *p* < 0.001). Trials with a low risk of bias had greater placebo responses (q1 = 5.47; I2 = 87.08; *p* = 0.02). The data from the present meta-analysis, including 1459 patients with clinical pain, suggest that placebo responses contribute significantly to the pain reduction observed in cannabinoid randomized clinical trials. The magnitude of the improvements in the placebo group was moderate to high and represents clinically relevant pain relief [141]. Special attention given to trials using cannabinoids with positive reports irrespective of scientific results may influence regulatory decisions, clinical practice, and ultimately patient access to cannabinoids for pain relief [141]. We must not neglect the psychotropic adverse effects of these substances.

During PE, by stimulating the ECS, we can observe analgesia, decreased anxiety and depression, euphoria, enhanced cognition, and many others effects [5]. Both acute and chronic exercise can improve cognitive performance [142,143], whereas psychological stress is known to impair cognitive functions [144,145]. During PE, increased AEA levels might be one of the key elements in the exercise-induced increment in brain-derived neurotrophic factor (BDNF) levels [96]. Moreover, high AEA levels during recovery might contribute to the return of BDNF levels to baseline [96]. BDNF produces beneficial effects on cognition through its ability to enhance neurogenesis, synaptic plasticity, and long-term potentiation, the basis of learning [146]. Specifically, blocking the CB1Rs in rats’ brains has been shown to diminish the BDNF increase following exercise, demonstrating a link between AEA binding and BDNF increase [147]. Decreased BDNF levels have been reported in people under great psychological stress [148].

PA increases serotonin in the brain, which is important for emotional processing and serves memory functions in the hippocampus [149,150,151]. PA has been shown to lead to an eCB-induced dopamine boost from the nucleus accumbens [96,152] and also increase dopamine levels in the striatum, hypothalamus, midbrain, and brainstem in various animal studies [153], further supporting the beneficial effects of exercise on memory and mood. Increases in serotonin and dopamine seem to modulate fatigue upon prolonged exercise. Exhaustion appears to set in when dopamine levels start to drop while serotonin levels are still elevated [154]. 

**Figure 3:**
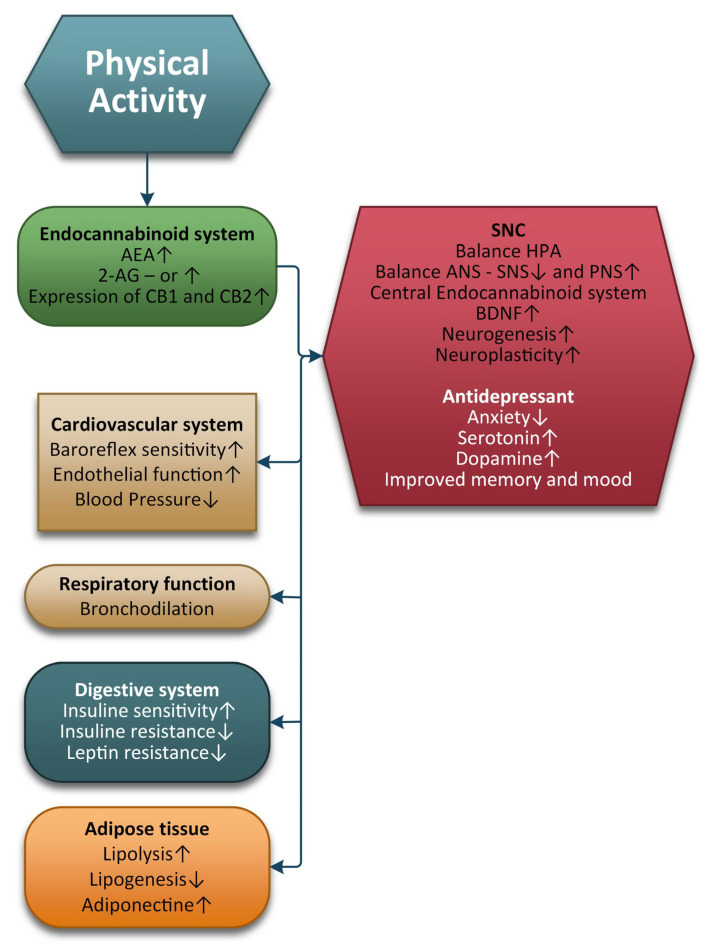
Basic changes in the endocannabinoids in multiple organs in response to physical exercise.

Figure 3 shows the basic changes in the endocannabinoids in multiple organs in response to physical exercise.

## 4. Conclusions

A growing body of evidence strongly indicates interplay between PE and the ECS, both centrally and peripherally. The ECS has an important role in controlling motor activity, cognitive functions, nociception, emotions, memory, and synaptic plasticity. The close interaction of the ECS with dopamine shows that they have a function in the brain’s reward system. Activation of the ECS also produces anxiolysis and a sense of wellbeing as well as mediates peripheral effects such as vasodilation and bronchodilation that may play a contributory role in the body’s response to exercise. Finally, the ECS may play a critical role in inflammation, as they modulate the activation and migration of immune cells as well as the expression of inflammatory cytokines.

Training can decrease systemic oxidative stress and it also has a positive impact on antioxidant defenses by increasing the expression of antioxidant enzymes.

PE is associated with reduced resting heart and respiratory rates and blood pressure; improved baroreflex, cardiac, and endothelial functions; increased skeletal muscle blood flow; increases blood flow to the brain; and reduced risk of stroke. PE also prevents age-associated reductions in brain volume, and is protective against the progression of various neurodegenerative disorders, cardiovascular diseases, obesity, metabolic syndrome, and type 2 diabetes mellitus

Physical activity restores a balance between the sympathetic and parasympathetic systems, ensuring the harmonious functioning of the autonomic nervous system. During PE, the activation of vagal afferents via TRP channels by the ECS produces stimulation of the PNS, which can activate the cholinergic anti-inflammatory pathway, and this can be considered a therapeutic strategy for reducing chronic inflammation and preventing many chronic diseases.

PE is considered a valuable non-pharmacological therapy that is an immediately available and cost-effective method with many health benefits, one of them being the activation of endogenous cannabinoids to reduce stress and anxiety and improve wellness.

## Data Availability

Not applicable.
